# Cost-effectiveness analysis of imaging surveillance in stage II and III extremity soft tissue sarcoma: an Australian perspective

**DOI:** 10.1186/s12962-020-0202-7

**Published:** 2020-02-03

**Authors:** Susie Bae, Jonathan Karnon, Glenis Crane, Taryn Bessen, Jayesh Desai, Phillip Crowe, Susan Neuhaus

**Affiliations:** 1 Peter MacCallum Cancer Centre, 305 Grattan St, Melbourne, VIC 3000 Australia; 2 Sir Peter MacCallum, Department of Oncology, The University of Melbourne, Parkville, VIC 3010 Australia; 3 College of Medicine and Public Health, Flinders University, 1 Flinders Dr, Bedford Park, SA 5042 Australia; 4 The University of Adelaide, Adelaide, SA 5005 Australia; 5 Department of Radiology, Royal Adelaide Hospital, Adelaide, SA 5000 Australia; 6 Prince of Wales Hospital, Sydney, 320-346 Barker St, Randwick, NSW 2031 Australia

**Keywords:** Soft tissue sarcoma, Cost-effectiveness, Imaging surveillance, Disease recurrence, Local recurrence, Distant recurrence, Pulmonary metastases, Metastasectomy

## Abstract

**Background:**

Surveillance imaging is used to detect local and/or distant recurrence following primary treatment of localised soft tissue sarcoma (STS), however evidence supporting optimal surveillance modality or frequency is lacking. We used prospectively collected sarcoma data to describe current surveillance imaging practice in patients with AJCC stage II and III extremity STS and evaluate its cost-effectiveness.

**Methods:**

From three selected Australian sarcoma referral centres, we identified patients with stage II and III extremity STS treated between 2009 and 2013. Medical records were reviewed to ascertain surveillance imaging practices, including modality, frequency and patient outcomes. A discrete event simulation model was developed and calibrated using clinical data to estimate health service costs and quality adjusted life years (QALYs) associated with alternative surveillance strategies.

**Results:**

Of 133 patients treated for stage II and III extremity STS, the majority were followed up with CT chest (86%), most commonly at 3-monthly intervals and 62% of patients had the primary site imaged with MRI at 6-monthly. There was limited use of chest-X-ray. A discrete event simulation model demonstrated that CT chest screening was the most cost effective surveillance strategy, gaining additional QALYs at a mean incremental cost of $30,743. MRI alone and PET-CT alone were not cost-effective, whilst a combined strategy of CT + MRI had an incremental cost per QALY gained of $96,556.

**Conclusions:**

Wide variations were observed in surveillance imaging practices in this high-risk STS cohort. Modelling demonstrated the value of CT chest for distant recurrence surveillance over other forms of imaging in terms of cost and QALYs. Further work is required to evaluate cost-effectiveness in a prospective manner.

## Background

Soft tissue sarcomas (STS) are rare malignant tumours arising from mesenchymal cells, which predominantly occur at the extremities [1]. In 2009, the incidence rate of STS was reported to be 6.12 per 100,000 Australian population, significantly increased by more than 50% from that in 1982 [2]. Local treatment with surgery with or without radiotherapy is the mainstay treatment for localised disease with the use of systemic therapy reserved for selected subtypes to optimise the chance of cure [3]. Surveillance following completion of primary treatment aims to identify local or distant recurrence at a stage when surgical intervention may improve overall survival or enable further limb conservation [4–11]. In particular, early identification and treatment of oligometastatic lung metastases has been shown to improve overall survival [6–11].

Evidence supporting international guidelines on optimal STS follow-up is poor [3, 12, 13]. Surveys demonstrate wide variation in surveillance imaging practices within the sarcoma specialist community [14–16]. In recent decades, new imaging modalities, such as magnetic resonance imaging (MRI) and positron emission tomography (PET) have become widely available. However, the relative merits of these imaging modalities in surveillance, in comparison to X-ray or CT remains to be defined. Increased consumer demand for imaging must be balanced with rising health care expenditure and the risks of over-investigation, increased radiation exposure and patient anxiety [1, 17, 18].

Most available evidence for optimal interval and modality of imaging surveillance comes from single centre experiences, which report conflicting results on superiority of CT chest over chest X-ray in detecting lung metastases, amenable for salvage metastastectomy [19–23]. Some have supported the role of surveillance MRI for early detection of asymptomatic local recurrence, whilst others have advocated the less intensive surveillance with chest X-ray and patient education about examination of the site of surgery with no detrimental effect on overall survival [24–27]. The ideal ‘gold-standard’ method to develop evidence-based practice would be to conduct a large multi-centre randomised controlled trial (RCT) to examine the surveillance imaging practice of varying modality and frequency. However, RCTs are costly, and may take many years to complete to reach statistical and clinical significance. A novel strategy to develop evidence-based guidelines is to predict the costs and utility of alternative surveillance imaging strategies in STS patient population using a model-based cost effectiveness analysis [28–31].

Our study was designed to utilise an Australian sarcoma database to describe current surveillance imaging practices in a high-risk patient cohort with localised extremity STS and assess cost-effectiveness using a discrete event simulation model.

## Methods

Data from three Australian sarcoma services were used to describe surveillance pathways and outcomes. A published cost-effectiveness model of alternative surveillance strategies for early breast cancer patients was adapted to replicate the patterns of recurrent cancer and death in patients diagnosed with high-risk localised extremity STS [32]. The model was populated using published evidence on progression of localised extremity STS, costs and health-related quality of life weights. Primary data was used to calibrate the model by comparing predicted and observed survival associated with observed surveillance pathways. This study was approved by the Human Research Ethics Committee at each site.

### Patient population

Patients were 18 years and older with American Joint Committee on Cancer (AJCC) Stages II and III (Grade 2 or 3, T1a to T2bN0M0) extremity STS, diagnosed between 1 January 2009 and 31 December 2013 and managed with curative-intent treatment. Patients’ follow-up surveillance imaging data were collected from medical records. To minimise heterogeneity within the cohort, sarcoma subtypes of extraosseous Ewing sarcoma, extraosseous osteosarcoma, and rhabdomyosarcoma were excluded.

### Model structure

**Fig. 1:**
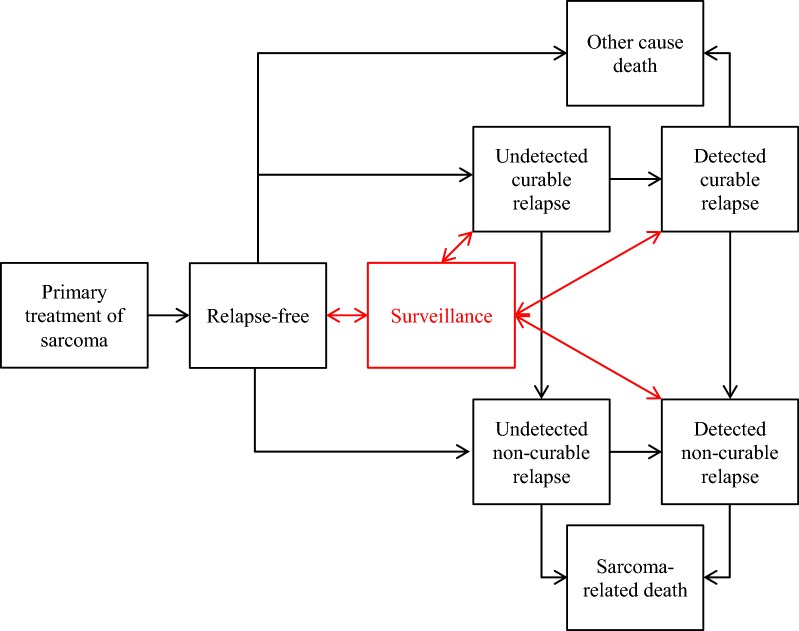
Sarcoma model structure

The cost-effectiveness model was implemented as a discrete event simulation due to the use of individual level patient data to populate and calibrate the model (Fig. 1). Patients enter the model disease-free following surgery of the primary tumour, but remain at risk of developing either local or oligometastatic recurrence. Recurrence may be detected by imaging surveillance or clinically. Detection of recurrence is assumed to result in treatment that reduces the risk of further disease progression. Following oligometastasis, patients are at risk of polymetastases. Patients with polymetastases are assumed to die from causes related to sarcoma. Patients may die from causes unrelated to sarcoma prior to the development of polymetastases.

The model represents the effects of alternative imaging-based surveillance pathways through the earlier detection of local and metastatic recurrence. Alternative forms of surveillance can be applied, including MRI (to detect local recurrence), CT scans (to detect metastatic recurrence) and PET scans (to detect both forms of recurrence). Patients with recurrence undergoing surveillance imaging move to a detected state (true positive imaging result) or move back to the same undetected state (false negative result). Patients without recurrence incur costs associated with further diagnostic tests if a false positive test result is returned.

### Model inputs

Initial ranges for model input parameter values were estimated from published literature and consensus discussion between the clinical and health economist authors of the paper. These estimates were further explored and consolidated at Australia and New Zealand Sarcoma Association (ANZSA) Scientific Advisory Committee Meeting and were thought to be consistent with the latest literature. Valid (or convergent) combinations of input values for the clinical parameters (disease progression, imaging test characteristics and clinical presentation probabilities) were identified using a calibration process in which model outputs were compared with 5- and 10-year cumulative metastatic disease and mortality probabilities for the observed clinical cohort, were estimated using the Sarculator^®^ (stratified prognosis of patients with high-risk STS) tool based on the baseline characteristics of the observed patient cohort [33].

The calibration process involved sampling sets of input parameter values from ranges for each clinical input parameter, which were based on literature. The cost-effectiveness model was run for each sampled set of parameter values, following 2000 hypothetical ‘patients’ from the time of diagnosis to the time of death. Each of the 2000 hypothetical patients was randomly assigned the baseline characteristics of one of the 133 patients in the observed cohort of patients, including the sampled patient’s observed surveillance pathway (i.e. the timing and type of surveillance received).

A sampled set of input parameter values was defined as convergent if the model predicted 5- and 10-year cumulative metastatic disease and mortality probabilities that were within the 95% confidence interval of the observed values. Calibration continued until 1000 sets of convergent input parameters were identified (Table 1) [28, 34–39].

**Table 1:** Parameter values

Parameter	Minimum	Maximum	Source
Disease-free to undetected local recurrence (5 year probability)	0.15	0.3	Guadagnolo et al. [34]
Disease-free to undetected oligometastasis (5 year probability)	0.3	0.45	Guadagnolo et al. [34]
Undetected to clinically detected local recurrence (1 year probability)	0.6	0.9	Expert opinion
Undetected to clinically detected oligometastasis (1 year probability)	0.5	0.8	Expert opinion
Undetected local recurrence to oligometastasis (1 year probability)	0.12	0.2	Expert opinion (based on detected local recurrence parameter values)
Clinically detected local recurrence to oligometastasis (1 year probability)	0.075	0.12	Daigeler et al. [35]; Guadagnolo et al. [34]; Whooley et al. [28]
Image detected local recurrence to oligometastasis (1 year probability)	0.075	0.12	Daigeler et al. [35]; Guadagnolo et al. [34]; Whooley et al. [28]
Undetected oligometastasis to polymetastases (1 year probability)	0.4	0.6	Expert opinion (based on detected oligometastases parameter values)
Detected oligometastasis to polymetastasis (1 year probability)	0.2	0.3	Welter et al. [36]
Polymetastasis to sarcoma-related death (1 year probability)	0.2	0.3	Whooley et al. [28]; Daigeler [35]
CT sensitivity	0.95	1	Christie-Large et al. [37]
CT specificity	0.9	1	Christie-Large et al. [37]
MRI sensitivity	0.87	1	Park et al. [38]
MRI specificity	0.65	0.79	Park et al. [38]
PET sensitivity	0.82	1	Bastiaannet et al. [39]
PET specificity	0.77	0.94	Bastiaannet et al. [39]
Proportion with metastatic disease at 5 years	0.25	0.32	Sarculator^®^
Proportion with metastatic disease at 10 years	0.28	0.36	Sarculator^®^
Mortality at 5 years	0.23	0.30	Sarculator^®^
Mortality at 10 years	0.32	0.40	Sarculator^®^

### Model analysis

The calibration model was adapted to apply cost and health-related quality of life (utility) weights to events experienced within the model (Table 2) [40]. The cost parameters were based on consultations, tests and procedures associated with each health state, as defined by the clinical authors, to which Medicare Benefits Schedule (MBS) item numbers were applied [41]. The utility input parameter values were derived from the published literature [32, 41]. The cost estimation of surgical procedures for recurrence did not take into account for post-operative complications and other ancillary costs such as rehabilitation.

**Table 2:** Utility cost and value

	Costs ($AUD)	Source from Medicare Benefits Schedule (MBS [33])	Utility value [31]
Disease-free state			0.832
CT surveillance (per visit)	340	MBS item 56,107	
MRI surveillance (per visit)	867	MBS item 15,559	
PET surveillance (per visit)	999	MBS item 61,646	
Undetected local recurrence			0.752
Undetected oligometastasis			0.655
Detected local recurrence (year 1)	7501	50% myocutaneous flap (MBS item no. 45,006) + 4 bed-days = $1038 + 4 × $1038 = $5190 50% free tissue transfer (MBS 45,564) + 7 day LoS = $2546 + 7 × $1038 = $9812	0.655
Detected local recurrence (year 2)	3043	Annual cost: Three consultations (MBS 104), chest X-rays (MBS 58,506) and MRIs = 3 × ($86.85 + $60.75 + $867)	0.752
Detected oligometastasis (year 1)	9945	Lung wedge resection (MBS 38,440) + pneumonectomy or lobectomy or segmentectomy (MBS 38,438) + 7 day LoS = $1147 + $1532 + 7 × $1038	0.655
Detected oligometastasis (year 2)	3258	Annual cost: Three consultations and full-body PET scans = 3 × ($86.85 + $999)	0.655
Detected polymetastasis (year 1)	63,284	Reported expected lifetime cost divided by 3 year expected survival [40]	0.443

1 bed-day cost = Commonwealth minimum benefit for single room accommodation (http://www.health.vic.gov.au/feesman/fees1.htm)

The final model was set-up to analyse a non-imaging (‘baseline surveillance’) option and three surveillance imaging strategies (MRI, CT and PET). The same imaging pathway was applied to all three imaging options: each 3 months for 2 years post-primary treatment, followed by 6 months until 5 years post-primary treatment, followed by annual imaging beyond 5 years.

For each of the evaluated surveillance scenarios, the same generated cohort of 2000 ‘patients’ was run through the model for each of the 1000 sampled sets of convergent input parameter values. The model time horizon was the remaining lifetime of the patient cohorts. Model outputs included discounted costs (health state costs, costs of surveillance and total costs), the percentage of ‘patients’ dying due to sarcoma, undiscounted life expectancy and discounted QALYs. Costs and QALYs were discounted at 5% per annum.

Two scenario analyses were undertaken with respect to the cost and utility input parameter values. In the analysis designed to favour surveillance options that only detect metastatic disease, costs associated with detected local recurrence and metastatic disease are increased and decreased by 25%, respectively, and the utility differences between the disease-free state and detected local recurrence and metastatic disease are decreased and increased by 50%, respectively. In the analysis designed to favour strategies that detect both local recurrence and metastatic disease, the opposite increases and decreases are applied to the cost and utility values.

Mean incremental cost-effectiveness ratios (ICERs) were estimated between the alternative surveillance imaging strategies. A probabilistic sensitivity analysis generated probabilities that each strategy is most cost-effective at alternative assumed monetary values for the gain of additional QALYs.

## Results

Baseline characteristics of patient population and treatments received are summarised in Table 3.

**Table 3:** Baseline characteristics of final study cohort

Patients demographics	Total	PMC	RAH	POW
	(N = 133)	(N = 92)	(N = 22)	(N = 19)
Mean age at diagnosis, years	55	53	61	63
Age range, years	19–90	19–90	19–90	27–80
Female to male	01:01.2	01:01.5	1.1:1	02:01
Stage				
II	53 (40%)	40	6	7
III	77 (58%)	49	20	8
Unknown^a^	3 (2%)	3	0	0
Sarcoma subtypes				
Undifferentiated pleomorphic sarcoma	53	39	8	6
Liposarcoma	26	15	5	6
Synovial sarcoma	19	18	1	0
Leiomyosarcoma	12	6	4	2
Other	23	14	4	5
Treatment breakdown				
Surgery alone	30	4	14	12
Surgery and radiotherapy^b^	95	86	7	2
Surgery and chemotherapy^c^	0	0	0	0
Surgery, radiotherapy, and chemotherapy^d^	8	2	1	5

^a^Localised soft tissue sarcomas with no evidence of metastasis and resectable but missing staging information

^b^Radiotherapy delivered in neoadjuvant or adjuvant setting

^c^Chemotherapy delivered in neoadjuvant or adjuvant setting

^d^Radiotherapy and chemotherapy delivered in neoadjuvant or adjuvant setting, concurrently or in sequence

### Surveillance imaging frequency and modality (observed patient cohort)

Three-monthly chest imaging was the most commonly adopted follow-up frequency in the first 2 years (n = 53, 46%). The rest were followed up between 4 and 6 monthly. Of 127 patients who commenced surveillance imaging, 86% (n = 110) were imaged with CT scan. Twelve patients (9%) underwent alternating chest X-ray and CT chest. Only three patients were followed up with chest X-ray alone. Two patients had no chest surveillance.

MRI was used to follow-up the primary site in 62% of patients (n = 79) at a variable time intervals between 3 and 6 months in the first 2 years. Ultrasound and CT scan were used sporadically to detect local recurrence in seven and three patients respectively. Twenty-six patients had PET-CT alone at least once during their surveillance period (20%). Overall, there was no consistent practice among clinicians or across the three institutions with regards to imaging frequency and modality.

### Treatment outcomes (observed patient cohort)

40 patients (30%) developed disease recurrence after a median follow-up of 29.9 months (range 0.3–67.7 months). 12.0% (n = 16) had local recurrence alone, 21.1% (n = 28) had distant recurrence, with the majority developing pulmonary metastases alone (n = 22, 78.6%). Six patients (4.5%) had both local and distant recurrences.

### Cost-effectiveness analysis

The cost-effectiveness model simulated the health service costs and QALYs that would be experienced by the observed cohort of patients with high-grade STS, for alternative surveillance strategies (Table 4). In the base case (no surveillance), the model predicted that 49% of the cohort is expected to die as a result of developing polymetastatic disease. Mean, undiscounted life expectancy across the whole cohort was 19.6 years. Discounted at 5% per annum, the expected mean QALY gain is 7.5 QALYs and expected costs related to the treatment of sarcoma are $42,483 per patient.

**Table 4:** Base case results

	Treatment costs	Surveillance costs	Total costs	Pr (sarcoma death)	QALYs	ICER
No surveillance	$42,483	$0	$42,483	0.426	7.50	
CT	$44,357	$4032	$48,389	0.402	7.69	$30,743
MRI	$39,266	$8068	$47,334	0.426	7.48	Dominated
CT + MRI	$44,202	$10,654	$54,857	0.393	7.76	$96,556
PET	$43,988	$25,567	$69,555	0.394	7.75	Dominated

CT screening was the least costly surveillance strategy, costing an expected $4032 per patient over their remaining lifetime. As a result of CT surveillance and earlier detection of metastatic disease, the costs of treating sarcoma recurrence and progression increased slightly to $44,357 to generate an aggregate lifetime cost per patient of $48,389. The CT alone strategy had an expected increment of 0.19 QALYs per patient, which generated an incremental cost per QALY gained of $30,743 per QALY gained compared to a no surveillance strategy.

Adding MRI to CT screening increased surveillance costs significantly, to $10,654 per patient. QALY gains increased to 7.76, an incremental gain of 0.07 QALYs, which resulted in an incremental cost per QALY gained of $96,556 compared to 6 monthly CT screening alone.

PET-CT surveillance has a theoretical advantage of enabling early detection of both local recurrence and metastatic disease with a single imaging modality, but it was marginally less effective than the combined CT + MRI option. It was also significantly most costly, which resulted in PET-CT being dominated (costing more and gaining fewer QALYs) by the CT + MRI option.

The results of the scenario analyses show that the use of cost and utility values that are more favourable to strategies that only detect metastatic disease decreased the ICERs associated with the CT screening options, with the more frequent CT screening option becoming the most cost-effective strategy (Table 5). In the scenario analysis favouring options able to detect both local recurrence and metastatic disease, the ICER for low frequency CT surveillance increases significantly to over $75,000 per QALY gained. The ICER for higher versus lower frequency CT surveillance decreases, but the ICER for the ICER for higher frequency CT surveillance versus no surveillance increases to almost $50,000 per QALY gained. The CT + MRI strategy becomes more attractive, whilst PET remains a dominated strategy.

**Table 5:** One-way sensitivity analysis

	Treatment costs	Surveillance costs	Total costs	Pr (sarcoma death)	Life years	QALYs	ICER
Scenario analyses designed favour surveillance strategies that detect only metastatic disease							
No surveillance	$35,022	$0	$35,022	0.43	19.57	7.67	
Low freq. CT	$35,260	$3184	$38,444	0.41	20.07	7.81	$24,483
CT	$35,796	$4031	$39,827	0.40	20.33	7.89	$16,335
CT + MRI	$36,171	$10,654	$46,825	0.39	20.55	7.95	$129,501
PET	$36,056	$25,566	$61,622	0.39	20.52	7.93	Dominated
Scenario analyses designed favour surveillance strategies that detect local recurrence and metastatic disease							
No surveillance	$49,864	$0	$49,864	0.43	19.57	7.25	
Low freq. CT	$51,885	$3184	$55,069	0.41	20.07	7.31	$75,672
CT	$52,810	$4031	$56,841	0.40	20.33	7.39	$24,967
CT + MRI	$52,113	$10,654	$62,766	0.39	20.55	7.47	$72,755
PET	$51,803	$25,566	$77,369	0.39	20.52	7.46	Dominated

**Fig. 2:**
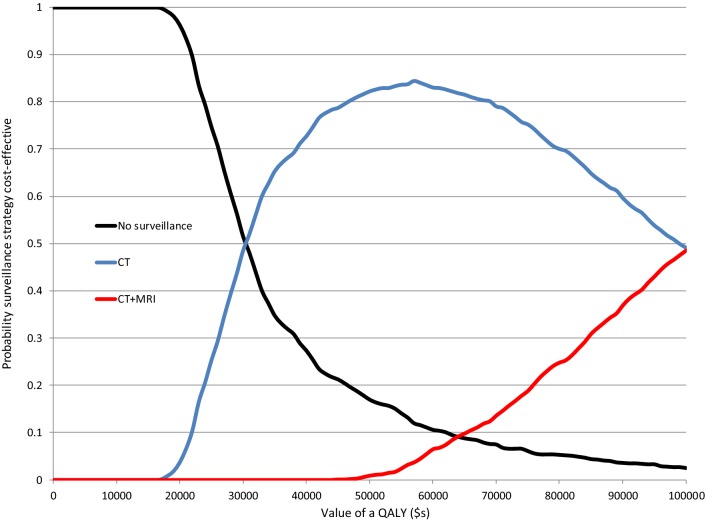
Cost-effectiveness acceptability curves

The outputs from a probabilistic sensitivity analysis are presented in the form of a cost-effectiveness acceptability curve in Fig. 2, which shows that both MRI and PET-CT strategies have a zero probability of being cost-effective no matter what monetary value is attached to the gain of additional QALYs. At a monetary value of $30,000 per QALY gained, the probability of CT surveillance being cost-effective is 49%, and at $50,000 the probability is around 82%. Beyond $50,000, the probability of CT + MRI being cost-effective starts to rise, reaching a probability of 49% at a monetary value of $100,000 per QALY gained.

**Fig. 3:**
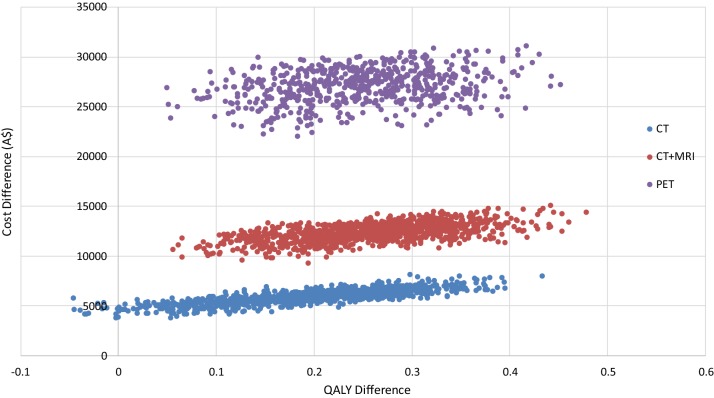
Cost-effectiveness plane

Figure 3 presents the results of the probabilistic sensitivity analysis in the form of a cost-effectiveness plane, which shows the separation of the cost differences (to a no surveillance option) and the overlapping QALY differences between the surveillance options.

## Discussion

Evidence for surveillance following primary soft tissue sarcoma treatment is scarce and survival benefits and cost-effectiveness has not been established [8, 42, 43]. The purpose of surveillance imaging is to detect disease recurrence early enough to allow for curative intervention and reduced disease-specific mortality. Current surveillance practices are widely variable and existing guidelines from the National Comprehensive Cancer Network and the European Society for Medical Oncology are based on expert consensus [3, 13]. (Table 6).

**Table 6:** Differences in recommended surveillance imaging between NCCN and ESMO guidelines

	NCCN, Version 1 2018 [3]	ESMO, 2018 [13]
Frequency	Stage 1: every 6–12 months	Low-grade STS: every 4–6 months in the first 3–5 years, then annually
	Stage II/III: every 3–6 months for 2–3 years, then every 6 months for next 2 years, then annually	Intermediate/high-grade STS: Every 3–4 months in the first 2–3 years; then twice a year up to the fifth year and once a year thereafter.
Modality	Primary site: MRI with or without contrast and/or CT with contrast or ultrasound for small, superficial lesions by an experienced ultrasonographer	Primary site: clinical assessment
	Chest: CT chest or chest X-ray	Chest: chest X-rays or CT chest at longer intervals

Variability in frequency and modality of follow-up is widely reported in the literature. A literature review by Goel et al. identified 54 different surveillance strategies of 5-year surveillance protocols for postoperative, low- to high-grade extremity STS covering years 1982 to 2003 in 34 published studies [29]. Clinical examination and chest X-ray were the most commonly used methods. Our study showed marked variability in post-operative surveillance across three Australian sarcoma services and between sarcoma specialists, however with infrequent use of chest X-ray. This may reflect easier access to more sophisticated imaging in Australia including the prevalent use of CT-PET.

High quality evidence to guide optimal surveillance is limited. Increased frequency of surveillance may lead to earlier detection of local or distant recurrence and improved survival. However, increased frequency also increases cost and may not be the most effective use of resources. Evidence for survival benefit for early detection of recurrence is lacking [5, 44]. The only available randomised study comes from a large Indian specialist cancer centre, where 500 non-metastatic sarcoma patients were randomised to four different surveillance protocols to determine non-inferiority of chest X-ray to CT scan and of less frequent (6-monthly) to more frequent (3-monthly) group [45]. In the updated analysis with a median follow up of 81 months, Puri et al. concluded that less intensive follow-up regimen with chest X-ray at 6-monthly intervals and patient education regarding self-examination of the primary sarcoma site detected the majority of distant and local recurrences without deleterious effects on overall survival [25]. The role of chest X-ray and physical examination should be explored in the Australian setting as adequate modality of surveillance.

Similar to our study design, Royce et al. used a computer simulation model to analyse the most cost-effective surveillance strategy for distant recurrence in a stage II and III extremity STS cohort [31]. Four different surveillance strategies were assessed, including ‘watchful waiting’ with no imaging, chest X-ray, CT chest or PET/CT. They concluded that optimal surveillance should be individualised based on patients’ risks for disease recurrence with CT chest as a preferred modality for imaging high-risk patients for distant recurrence and chest X-ray or CT chest at a less frequent interval as a preferred option for low-risk patients.

Our study has several limitations worth noting. Firstly, the model is based on a small number of patients from heterogeneous population of soft tissue sarcomas from three selected sarcoma referral centres. Given the rarity of individual sarcoma histology subtype, heterogeneity inevitably complicates studies of this disease group. In addition, chest X-ray was not included as a surveillance option due to low utilisation in our patient population and physical examination as a modality to detect local recurrence was not explored in this analysis, rather we concentrated more on the use of surveillance imaging. Despite these limitations this study is the first of its kind reflecting on the ‘real-world’ practice in the Australian setting and utilising patient data to generate a computer simulation model.

The soaring cost of health care is a well-recognised problem worldwide. Surveillance following primary cancer treatment must balance gains in survival with societal willingness to expend health care resources and cost effectiveness. Our study suggests that CT surveillance of chest is the most cost-effective surveillance option and frequent use of MRI and PET is not supported by its significant cost burden.

## Conclusions

This study confirms wide variation in surveillance practices amongst Australian sarcoma specialists within the high-risk STS cohort. Relatively intense imaging follow-up strategies were practiced at Australian sarcoma referral centres. For patients with high-risk extremity STS, more frequent surveillance for distant disease recurrence with CT scan appears cost-effective at an acceptable gain in QALY. Further work is required to evaluate cost-effectiveness in a prospective manner and to stratify surveillance against risk.

## Data Availability

The datasets used for the analysis in the study can be made available from the corresponding author upon reasonable request.

## Abbreviations

AJCC: American Joint Committee on Cancer
ANZSA: Australia and New Zealand Sarcoma Association
CT: Computed tomography
ICER: Incremental cost-effectiveness ratios
MRI: Magnetic resonance imaging
MBS: Medicare Benefits Schedule
QALY: Quality adjusted life years
PET: Position emission tomography
STS: Soft tissue sarcomas
